# Estimating the Undetected Burden of Respiratory Syncytial Virus Hospitalizations in Adults Through Capture–Recapture Methods

**DOI:** 10.1111/irv.13299

**Published:** 2024-05-03

**Authors:** Amanda C. Howa, Yuwei Zhu, Dayna Wyatt, Tiffanie Markus, James D. Chappell, Natasha Halasa, Christopher H. Trabue, William Schaffner, Carlos G. Grijalva, H. Keipp Talbot

**Affiliations:** ^1^ Department of Health Policy Vanderbilt University Medical Center Nashville Tennessee USA; ^2^ Department of Biostatistics Vanderbilt University Medical Center Nashville Tennessee USA; ^3^ Department of Pediatrics Vanderbilt University Medical Center Nashville Tennessee USA; ^4^ Department of Medicine University of Tennessee College of Medicine Nashville Tennessee USA; ^5^ Department of Medicine Vanderbilt University Medical Center Nashville Tennessee USA; ^6^ Department of Biomedical Informatics Vanderbilt University Medical Center Nashville Tennessee USA

**Keywords:** capture–recapture, hospitalizations, respiratory syncytial virus, RSV

## Abstract

**Introduction:**

Traditional surveillance systems may underestimate the burden caused by respiratory syncytial virus (RSV). Capture–recapture methods provide alternatives for estimating the number of RSV‐related hospitalizations in a population.

**Methods:**

Capture–recapture methods were used to estimate the number of RSV‐related hospitalizations in adults in Middle Tennessee from two independent hospitalization surveillance systems during consecutive respiratory seasons from 2016–2017 to 2019–2020. Data from the Hospitalized Adult Influenza Vaccine Effectiveness Network (HAIVEN) and the Emerging Infections Program (EIP) were used. Annual RSV hospitalization rates were calculated using the capture–recapture estimates weighted by hospitals' market share divided by the corresponding census population.

**Results:**

Using capture–recapture methods, the estimated overall adult hospitalization rates varied from 8.3 (95% CI: 5.9–15.4) RSV‐related hospitalizations per 10,000 persons during the 2016–2017 season to 28.4 (95% CI: 18.2–59.0) hospitalizations per 10,000 persons in the 2019–2020 season. The proportion of hospitalizations that HAIVEN determined ranged from 8.7% to 36.7% of the total capture–recapture estimated hospitalization, whereas EIP detected 23.5% to 52.7% of the total capture–recapture estimated hospitalizations.

**Conclusion:**

Capture–recapture estimates showed that individual traditional surveillance systems underestimated the hospitalization burden in adults. Using capture–recapture allows for a more comprehensive estimate of RSV hospitalizations.

## 1 Introduction

Respiratory syncytial virus (RSV) is a common respiratory virus that generally causes mild disease but is also associated with severe disease in individuals in the extreme of ages—infants and older adults [1]. While the burden of RSV in children is better established, the burden in adults is not as well defined. Determining the burden of RSV infections is of great public health interest to inform and target preventive activities such as vaccination programs. With the recent approval of a RSV vaccine for older adults, it is of vital importance to use surveillance programs to determine RSV burden and monitor the impact of vaccination programs.

RSV‐associated symptoms are similar to those caused by other respiratory viruses, so definitive diagnosis requires laboratory confirmation [1, 2]. Additionally, elderly individuals with RSV may present with atypical symptoms such as confusion, anorexia, falls, or related complications, such as worsening of congestive heart failure or an exacerbation of chronic obstructive lung disease; therefore, making diagnosis based on clinical findings may be challenging in adults [2, 3]. Additionally, those with high‐risk or chronic medical conditions, especially cardiopulmonary disease, may be at higher risk of experiencing severe RSV‐related outcomes [4]. Historically, RSV has been a challenging pathogen to detect and notoriously difficult to culture. While previous RSV rapid antigen tests showed relatively good performance in children, they often performed less effectively in adults due to the requirement for substantial viral quantities to produce a positive test result. This is because older adults typically exhibit lower viral loads in their respiratory secretions [2]. Thus, highly sensitive molecular detection techniques, such as RT‐PCR, are currently the preferred diagnostic technique for detection of RSV infections [2]. Considering these factors, surveillance relying on clinician‐directed testing based on symptoms or clinical presentation may underestimate the prevalence of RSV disease.

When two or more independent hospital‐based surveillance systems operate concurrently in a geographic area, capture–recapture methods can use the data from those systems to obtain a more comprehensive estimate of the burden of hospitalizations [5]. Utilizing data from the Emerging Infections Program (EIP) and the Hospitalized Adult Influenza Vaccine Effectiveness Network (HAIVEN), we applied capture–recapture methods to calculate RSV‐related hospitalization rates for adults during consecutive respiratory seasons from 2016–2017 through 2019–2020 in Middle Tennessee.

## 2 Methods

### 2.1 Hospital‐Based Surveillance Systems

EIP and HAIVEN are two hospital‐based surveillance systems that operated concurrently in Middle Tennessee during four consecutive respiratory seasons (2016–2020). EIP is a collaborative program between the Centers for Disease Control and Prevention (CDC), state health departments, and academic institutions that conduct active laboratory and population‐based infectious disease surveillance. EIP identifies all patients hospitalized with any clinician‐ordered positive RSV laboratory test from specimens collected 14 days prior to or during hospitalizations through review of hospital laboratory results and infection control logs.

HAIVEN identified adult patients hospitalized with acute respiratory illness symptoms through electronic medical review. Research staff approached and consented patients with a new onset or worsening of respiratory symptoms within the 10 previous days and then collected nasal and oropharyngeal swabs, independent from clinician ordered testing. All collected HAIVEN specimens were tested by RT‐PCR for RSV. Enrollment occurred approximately 5 days per week but allowed enrollment within 48–72 h from admission allowing comprehensive enrollment of patients no matter day of admission. This analysis was restricted to periods of time when surveillance for HAIVEN and EIP overlapped.

EIP was determined by the VUMC, the State of Tennessee, and the CDC as public health surveillance, and HAIVEN was approved by the VUMC, Sterling, and CDC IRBs.

### 2.2 Study Population

The study's geographical catchment area encompassed Davidson County and its adjacent seven counties where EIP was active: Cheatham, Dickson, Robertson, Rutherford, Sumner, Williamson, and Wilson. Since not all hospitals participating in EIP were part of the HAIVEN surveillance, our analysis focused on three hospitals where both HAIVEN and EIP conducted surveillance simultaneously. Therefore, we restricted our analysis to three hospitals located in Nashville, Tennessee, where both EIP and HAIVEN were concurrently engaged in surveillance. EIP defined cases as hospitalized adults who had a positive laboratory‐confirmed RSV test result. These tests were ordered by a provider, as part of routine clinical care. Cases from HAIVEN were defined as adults hospitalized with an acute respiratory illness who enrolled in a prospective study and had a research specimen collected that tested positive in a research laboratory. HAIVEN collected and tested specimens independently from any routine clinical testing. Cases identified by both systems (i.e., overlapping) were identified by matching the following: first and last name, date of birth, county of residence, admission hospital, and date of admission. Four consecutive respiratory seasons were included in the analyses, 2016–2017, 2017–2018, 2018–2019, and 2019–2020, with each season defined as October 1 through April 30. During the 2019–2020 season, surveillance was interrupted by the COVID‐19 pandemic that started in March 2020.

### 2.3 Statistical Analyses

The capture–recapture estimation was calculated using Chapman's capture–recapture method, which is preferred over other capture–recapture methods when using small numbers (Figure S1). We used capture–recapture methods to estimate the number of people hospitalized with RSV (*N*) within each respiratory season for the three hospitals in our study by calculating $\hat{N}$. This was done by using the total number of hospitalizations each surveillance system, EIP and HAIVEN, found and matched the patients that overlapped between both systems. For the capture–recapture calculation, HAIVEN captured *n1* RSV hospitalizations and EIP captured *n2* RSV hospitalizations. The cases that both surveillance systems captured are labeled as *m2*. We used transformed logit 95% confidence intervals for the Chapman capture–recapture estimations based on small sample sizes. HAIVEN actively enrolled participants hospitalized with respiratory symptoms, regardless of clinician ordered testing, whereas EIP relied on results of clinician ordered testing.

The proportion of RSV hospitalizations detected individually by EIP and HAIVEN was calculated as the number of RSV hospitalizations detected by each surveillance system divided by the total number of RSV hospitalizations estimated by the capture–recapture method. Additionally, we used the capture–recapture estimates to compute RSV hospitalization rates using hospital market share data and census population estimates. Hospital market share represents the proportion of hospitalizations for acute respiratory illness recorded in surveillance hospitals out of all hospitalizations in the Middle Tennessee catchment area. Hospitalization data were procured from the Hospital Discharge Data System (HDDS) [6], a registry of all hospitalizations in Tennessee, which also includes demographic information and hospital discharge codes. The RSV hospitalization rates for Middle Tennessee were then calculated by weighting the capture–recapture estimates to account for the hospital market share data, and then divided by the corresponding census population estimates. RSV hospitalization rates were calculated by respiratory season and age group.

The capture–recapture analysis follows the assumption that the probability of capture by one surveillance system is independent of the probability of capture by another. Therefore, all patients regardless of day of admission would need to be eligible to participate in both surveillance systems. EIP collects data from every positive test all 7 days each week, so HAIVEN enrollment data was examined to explore if this assumption was fulfilled. For this, the number of participants enrolled in HAIVEN was divided by the number that were found eligible for the study by day of the week to determine if enrollment numbers were similar by day of the week of admission. Analyses were performed using Stata, version 16.1 (StataCorp LP, College Station, TX, USA) and SAS Enterprise Guide 8.1 (SAS Institute, Cary, NC, USA).

## 3 Results

### 3.1 Capture–Recapture Estimates of RSV Hospitalizations

**FIGURE 1:**
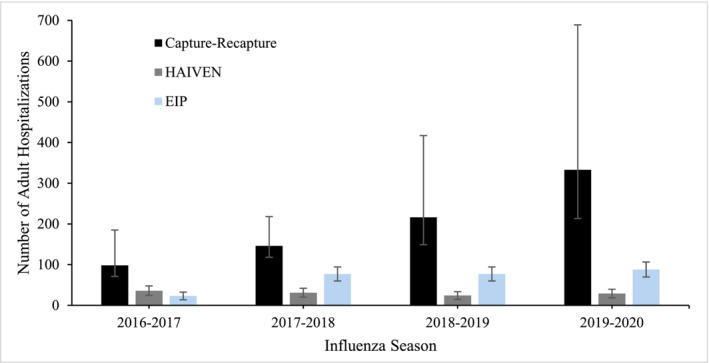
Unweighted adult RSV hospitalizations by capture–recapture estimates and surveillance systems for adult cases for three hospitals in Middle Tennessee across four respiratory seasons. Transformed logit confidence intervals were used for capture‐recapture, Poisson confidence intervals were used for EIP and HAIVEN. EIP, Emerging Infections Program; HAIVEN, Hospitalized Adult Influenza Vaccine Effectiveness Network.

Capture–recapture methods estimated that across the four respiratory seasons there were between 47 and 223 missed RSV‐associated hospitalizations of adults in the three hospitals (Table S1). The 2016–2017 respiratory season had the lowest number of RSV hospitalizations with 47 missed cases for a total of 98 (95% CI: 71–185) estimated hospitalizations. The highest number of missed RSV hospitalizations was during the 2019–20 respiratory season, which had an estimated 223 missed cases for a total of 333 (95% CI: 213–689) estimated hospitalizations among the three hospitals (Table 1 and Figure 1).

**TABLE 1:** Estimated capture–recapture hospitalizations of adults detected by concurrent surveillance systems across four respiratory seasons, in three hospitals in Middle Tennessee.

	2016–2017	2017–2018	2018–2019	2019–2020
HAIVEN	36	31	24	29
EIP	23	77	77	88
Overlapped	8	16	8	7
Capture–recapture	98	146	216	333

Abbreviations: EIP, Emerging Infections Program; HAIVEN, Hospitalized Adult Influenza Vaccine Effectiveness Network.

### 3.2 Proportion of Capture–Recapture Estimated Cases Captured by EIP and HAIVEN

The capture–recapture estimates were used to calculate the proportion of RSV hospitalizations of adults that EIP and HAIVEN identified (Table 2). EIP captured a higher proportion than HAIVEN in all seasons except during the 2016–2017 respiratory season when EIP detected 23.5% (95% CI: 15.1–31.9) and HAIVEN detected 36.7% (95% CI: 27.2–46.3) of estimated cases. EIP captured between 23.5% (95% CI: 15.1–31.9) of estimated cases in 2016–2017 and 52.7% (95% CI: 44.6–60.8) of estimated cases in 2017–2018. HAIVEN proportion of cases detected ranged between 8.7% (95% CI: 5.7–11.7) of estimated cases in 2019–2020 and 36.7% (95% CI: 27.2–46.3) of estimated cases in 2016–2017.

**TABLE 2:** Proportion^a^ of estimated capture–recapture hospitalizations of adults detected by surveillance system and respiratory season in three hospitals in Middle Tennessee.

	2016–2017		2017–2018		2018–2019		2019–2020	
	Proportion	95% CI	Proportion	95% CI	Proportion	95% CI	Proportion	95% CI
HAIVEN	36.7%	27.2–46.3	21.2%	14.6–27.9	11.1%	6.9–15.3	8.7%	5.7–11.7
EIP	23.5%	15.1–31.9	52.7%	44.6–60.8	35.6%	29.3–42.0	26.4%	21.7–31.2

Abbreviations: CI, confidence interval; EIP, Emerging Infections Program; HAIVEN, Hospitalized Adult Influenza Vaccine Effectiveness Network.

^a^ Proportion of hospitalization detected was calculated as the number of hospitalizations each surveillance system detected divided by the total number of hospitalizations estimated by the capture–recapture analysis.

### 3.3 Estimated RSV Hospitalization Rates in Middle Tennessee

RSV hospitalization rates were estimated using hospital market share data, which remained relatively stable over the four seasons (data not shown) and census population numbers. The total hospitalization rates based on the capture–recapture estimates ranged from 8.3 per 10,000 adults in 2016–2017 to 28.4 per 10,000 adults in 2019–2020 (Table 3). The analysis stratified by age groups showed the highest RSV‐related hospitalization rates were in the 65+ years age group, followed by the 50–64 years age group. The highest age‐stratified RSV hospitalization rate over the complete surveillance period was observed in the 2019–2020 season, when an estimated 90.9 hospitalizations per 10,000 persons occurred in the 65+ years age group.

**TABLE 3:** Estimated RSV hospitalization rates per 10,000 adults by age group in Middle Tennessee across four respiratory seasons based on capture–recapture estimates and market share.

	2016–2017		2017–2018		2018–2019		2019–2020	
	Rate per 10,000	95% CI	Rate per 10,000	95% CI	Rate per 10,000	95% CI	Rate per 10,000	95% CI
18–49 years	2.3	1.6–4.1	2.9	2.4–4.4	6.5	4.4–12.4	7.9	5.1–16.4
50–64 years	10.0	7.2–18.6	13.5	10.9–20.1	21.3	14.6–40.8	32.4	20.8–67.2
65+ years	27.4	19.7–51.3	39.6	31.6–58.5	62.9	43.4–121.4	90.9	58.3–188.7
Total	8.3	5.9–15.4	11.6	9.4–17.3	19.8	13.6–38.0	28.4	18.2–59.0

Abbreviation: CI, confidence interval.

### 3.4 Examining Assumptions of Capture–Recapture Estimation

The proportion of patients enrolled in HAIVEN by each day of week was relatively stable. Overall enrollment of eligible patients varied season to season and was lowest during the 2018–2019 season (28.83%) and highest during the 2016–2017 season (50.40%) (Table S2).

## 4 Discussion

We employed capture–recapture methods to amalgamate data from two surveillance systems, EIP and HAIVEN, and to estimate the rate of RSV‐related hospitalizations among adults in Middle Tennessee throughout four consecutive respiratory seasons. Recognizing that individual surveillance systems possess inherent limitations, we leveraged capture–recapture techniques to use data from two pre‐existing, geographically coinciding surveillance systems. This approach yielded an estimation of hospitalizations that did not rely solely on either system and found higher RSV‐related hospitalization rates than either individual surveillance system found.

EIP captures every RSV‐associated hospitalization with a positive test result but relies on clinician‐ordered testing so it will miss any hospitalized cases that were not tested specifically for RSV. As no RSV‐specific treatment is available, clinicians may not be inclined to always test for RSV if suspected. HAIVEN actively conducted surveillance independently of clinician‐ordered testing but may miss participants who were not approached and those who did not consent to participate. Compared with capture–recapture estimates, we calculated that neither surveillance system detected more than 52.7% of hospitalizations during any season indicating each system alone underestimated the number of RSV‐related adult hospitalizations. The underestimation of the RSV‐related hospitalization burden may be due to several issues, such as low awareness of RSV infections in adults, a lack of routine testing [7], indicating the importance of using multiple surveillance systems to properly identify the RSV hospitalization burden.

Previous studies have estimated a wide range of hospitalizations associated with RSV. Our estimates fall in the higher range of prior reports. The CDC's RSV‐Associated Hospitalization Surveillance Network (RSV‐NET) estimated that for the 2016–2017 the overall national rate of hospitalizations was 0.88 per 10,000 compared to our estimated rate of 8.3 per 10,000 for Middle Tennessee. For the 2017–2018 season, the CDC estimated the national hospitalization rate was 1.67 per 10,000 adults, where our estimated hospitalization rate was 11.6 hospitalization per 10,000. For the 2018–2019 season, our estimates were also higher at 19.8 hospitalizations per 10,000 compared to the CDC's estimated 1.16 per 10,000 hospitalizations. Additionally for the 2019–2020 season, our estimated hospitalization rate was the highest at 28.4 per 10,000 compared to the CDC's 1.42 per 10,000 hospitalizations [8]. The CDC's RSV surveillance uses the EIP RSV‐NET data but estimates the amount of undertesting for RSV in the clinic setting as a correction factor to determine rates of hospitalizations. Additionally, surveillance is limited to some states and settings and those may not be reflective of the local rates experienced in Middle Tennessee. Branche et al. conducted surveillance at three New York hospitals from 2017 to 2020 [9] and found lower RSV‐related hospitalizations ranging from 4.4 to 5.9 per 10,000 in adults ≥ 18 years of age. Similar to HAIVEN, Branche et al. reviewed hospital logs to identify patients meeting the case definition and obtained informed consent for RSV testing but did not conduct recruitment over the weekend. Unlike HAIVEN, which tested for multiple respiratory diseases, Branche et al. specifically tested for RSV and excluded any 24‐h observations and only included adults that were hospitalized for 24 or more hours. Branche et al. tested over 90% of the population that met their acute respiratory illness case definition and therefore very few individuals were missed due to clinician testing whereas in middle TN, fewer patients were clinically tested for RSV. The ideal surveillance system would involve testing every patient meeting the case definition, but in practice, our capture–recapture approach provides an alternate way to estimate the disease burden using available systems. While our estimates are higher than some reports, it is unclear if previous literature underestimated RSV‐related hospitalizations. These differences may have contributed to their smaller hospitalization rates compared to what we found. There may also have been potential differences due to differences in geographic locations. In an earlier study, Widmer et al. reported RSV‐related hospitalization rates of 15.0 per 10,000 in those above 50 years of age and 25.4 per 10,000 in those above 65 years of age in Middle Tennessee from 2006 to 2009 [10]. This study prospectively enrolled patients with an acute respiratory illness, similar to Branche, and likely underestimated cases due to the enrollment and consent process of their studies. The inclusion of EIP in the current study likely overcame the issues of approach and enrollment. Matias et al. used multiple regression modeling with virological surveillance and hospitalization data from 1997 to 2009 and found RSV‐related hospitalization rates of 6.4 per 10,000 in those of all ages, with higher hospitalization rates of 8.4 per 10,000 in those aged 65–74 years and 25.8 per 10,000 in those 75+ [11]. Zheng et al. used regression models to estimate the incidence of RSV‐related hospitalization in New York, New Jersey, and Washington from 2005 to 2014 and stratified by socioeconomic status and age. They determined cause specific hospitalizations through ICD‐9‐CM discharge information from the US State Inpatient Databases and estimated an incidence of 13 to 96 per 10,000 people aged greater than 65 years of age [12]. The different study periods of both Matias et al. and Zheng et al. add to the difficulty in comparing the wide range of estimated hospitalization rates to our capture–recapture estimates. Our hospitalization rates were highest in adults ≥ 65 years of age, supporting additional literature showing substantial burden in older adults and an increase in medically‐attended RSV with age [13]. Additional studies support these findings by showing higher RSV‐related morbidity and mortality among older adults and underly the importance of increased surveillance identifying and quantifying the RSV burden in adults [14, 15, 16, 17].

This study has limitations. Capture–recapture methodology has several assumptions that need to be met for the capture–recapture estimates to be valid. The estimates are valid if the probability of being captured by one surveillance system is independent of capture by the other surveillance system, the population remains stable and without significant migration during the study period, and the cases can be matched by both systems. The independence assumption is difficult to verify, however, to account for this we examined the proportion of people enrolled out of those found eligible for HAIVEN to ensure equal opportunity for being captured across every day of the week. While a stable population is also difficult to demonstrate, no mass migration events took place during the study period. The third assumption, that cases can be matched, was met as we were able to identify laboratory‐confirmed cases and match cases between surveillance systems. We do acknowledge uncertainty in our estimated numbers as reflected in wide confidence intervals for some estimates, especially when the number of overlapping cases is low, which is highlighted during the 2019–2020 season. Additionally, there may have been changes in provider testing practices during the surveillance period, which may have impacted EIP surveillance. Finally, our estimates were derived from surveillance systems that operated in Middle Tennessee and our findings may not be directly generalizable to other settings or populations.

Utilizing capture–recapture methods allowed us to obtain comprehensive estimates for the RSV burden in adults across four consecutive respiratory seasons in Middle Tennessee. We found that using one surveillance system underestimates the burden of RSV‐related hospitalizations, and using both surveillance systems together found a larger burden than either system alone. The capture–recapture approach can be useful to inform resource allocation and monitor the impact of RSV vaccination policies for older adults.

## Author Contributions

**Amanda C. Howa:** Writing – original draft; Writing – review and editing; Formal analysis. **Yuwei Zhu:** Formal analysis; Writing – review and editing. **Dayna Wyatt:** Writing – review and editing. **Tiffanie Markus:** Writing – review and editing. **James D. Chappell:** Writing – review and editing. **Natasha Halasa:** Writing – review and editing; Data curation. **Christopher H. Trabue:** Writing – review and editing. **William Schaffner:** Writing – review and editing. **Carlos G. Grijalva:** Conceptualization; Writing – review and editing. **H. Keipp Talbot:** Data curation; Writing – review and editing; Funding acquisition; Conceptualization.

## Conflicts of Interest

C.G.G. reports grants from Syneos Health, the National Institutes of Health, the Food and Drug Administration, the Agency for Health Care Research and Quality, and consultation fees from Merck. N.H. reports grants from Sanofi, Merck, and Quidell. The other authors declare no conflicts of interest.

### Peer Review

The peer review history for this article is available at https://www.webofscience.com/api/gateway/wos/peer‐review/10.1111/irv.13299.

## Supporting information


**Figure S1.** Capture–recapture estimation using data from two independent surveillance sources using Chapman’s method.
**Table S1.** Capture–recapture estimates using Chapman’s method. RSV hospitalizations of adults in three hospitals in Middle Tennessee across four respiratory seasons.
**Table S2.** The percent of participants enrolled out of those found eligible for the study, HAIVEN.

## Data Availability

The data are not publicly available due to privacy or ethical restrictions. The data that support the findings of this study are available on request from the corresponding author.
