# Supplementary material for: Estimating the Undetected Burden of Respiratory Syncytial Virus Hospitalizations in Adults Through Capture–Recapture Methods
Source: Influenza Other Respir Viruses. 2024 May 3;18(5):e13299. doi: 10.1111/irv.13299 (PMC11066857; doi:10.1111/irv.13299)
Supplement: Supplementary file 1 — Figure S1. Capture–recapture estimation using data from two independent surveillance sources using Chapman’s method. Table S1. Capture–recapture estimates using Chapman’s method. RSV hospitalizations of adults in three hospitals in Middle Tennessee across four respiratory seasons. Table S2. The percent of participants enrolled out of those found eligible for the study, HAIVEN. [file IRV-18-e13299-s001.docx]

**Supplemental Figure 1.** Capture-recapture estimation using data from two independent surveillance sources using Chapman’s method.

|  |  | Active enrollment and testing surveillance (HAIVEN) | |  |
| --- | --- | --- | --- | --- |
|  |  | Enrolled | Missed |  |
| Laboratory testing surveillance (EIP) | Enrolled | *a=m2* | *b* | *n2= (a+b)* |
|  | Missed | *c* | *z* |  |
|  |  | *n1= (a+c)* |  | $\hat{N}$ *=* $\frac{\left( n2+1 \right)\left( n1+1 \right)}{m2+1}-1$ |

**Supplemental Table 1.** Capture-recapture estimates using Chapman’s method. RSV hospitalizations of adults in three hospitals in Middle Tennessee across four respiratory seasons.

| a. 2016-2017   \|  \|  \| Active enrollment and testing surveillance (HAIVEN) \| \|  \| \| --- \| --- \| --- \| --- \| --- \| \|  \|  \| Enrolled \| Missed \|  \| \| Laboratory testing surveillance (EIP) \| Enrolled \| 8 \| 15 \| 23 \| \| Missed \| 28 \| 47 \|  \| \|  \|  \| 36 \|  \|  \| \|  \| $\hat{N}$ = 98 (95% CI: 71-185) \| \| \|  \| | b. 2017-2018   \|  \|  \| Active enrollment and testing surveillance (HAIVEN) \| \|  \| \| --- \| --- \| --- \| --- \| --- \| \|  \|  \| Enrolled \| Missed \|  \| \| Laboratory testing surveillance (EIP) \| Enrolled \| 16 \| 61 \| 77 \| \| Missed \| 15 \| 54 \|  \| \|  \|  \| 31 \|  \|  \| \|  \| $\hat{N}$ = 146 (95% CI: 118-218) \| \| \|  \| |
| --- | --- | --- | --- | --- | --- | --- | --- | --- | --- | --- | --- | --- | --- | --- | --- | --- | --- | --- | --- | --- | --- | --- | --- | --- | --- | --- | --- | --- | --- | --- | --- | --- | --- | --- | --- | --- | --- | --- | --- | --- | --- | --- | --- | --- | --- | --- | --- | --- | --- | --- | --- | --- | --- | --- | --- | --- | --- | --- | --- |
| c. 2018-2019   \|  \|  \| Active enrollment and testing surveillance (HAIVEN) \| \|  \| \| --- \| --- \| --- \| --- \| --- \| \|  \|  \| Enrolled \| Missed \|  \| \| Laboratory testing surveillance (EIP) \| Enrolled \| 8 \| 69 \| 77 \| \| Missed \| 16 \| 123 \|  \| \|  \|  \| 24 \|  \|  \| \|  \| $\hat{N}$ = 216 (95% CI: 149-417) \| \| \|  \| \|  \|  \| \| \|  \| | d. 2019-2020   \|  \|  \| Active enrollment and testing surveillance (HAIVEN) \| \|  \| \| --- \| --- \| --- \| --- \| --- \| \|  \|  \| Enrolled \| Missed \|  \| \| Laboratory testing surveillance (EIP) \| Enrolled \| 7 \| 81 \| 88 \| \| Missed \| 22 \| 223 \|  \| \|  \|  \| 29 \|  \|  \| \|  \| $\hat{N}$ = 333 (95% CI: 213-689) \| \| \|  \| |

HAIVEN, Hospitalized Adult Influenza Vaccine Effectiveness Network; EIP, Emerging Infections Program; CI, Confidence Interval

**Supplemental Table 2.** The percent of participants enrolled out of those found eligible for the study, HAIVEN

| Season | Sunday | CI | Monday | CI | Tuesday | CI | Wednesday | CI | Thursday | CI | Friday | CI | Saturday | CI |
| --- | --- | --- | --- | --- | --- | --- | --- | --- | --- | --- | --- | --- | --- | --- |
| 2016-2017 | 58.77% | 52.38-65.16 | 50.90% | 45.54-56.26 | 46.59% | 40.57-52.61 | 50.49% | 44.91-56.06 | 47.72% | 41.41-54.02 | 47.68% | 39.72-55.65 | 50.00% | 40.48-59.52 |
| 2017-2018 | 42.86% | 37.33-48.38 | 45.71% | 40.30-51.11 | 46.85% | 41.07-52.64 | 48.06% | 42.24-53.88 | 40.19% | 33.54-46.84 | 49.68% | 41.86-57.50 | 38.04% | 30.58-45.49 |
| 2018-2019 | 29.53% | 25.22-33.85 | 29.59% | 25.72-33.46 | 30.83% | 26.70-34.96 | 28.00% | 23.96-32.04 | 27.64% | 23.35-31.94 | 28.29% | 22.71-33.86 | 26.71% | 21.76-31.66 |
| 2019-2020 | 31.77% | 26.97-36.56 | 31.39% | 26.90-35.87 | 31.12% | 26.25-36.00 | 32.72% | 28.02-37.43 | 29.88% | 24.92-34.83 | 26.06% | 19.79-32.34 | 34.50% | 27.91-41.09 |
